# Coherent THz Hyper-Raman: Spectroscopy and Application in THz Detection

**DOI:** 10.3390/ma12233870

**Published:** 2019-11-23

**Authors:** Arianna Ceraso, Sen Mou, Andrea Rubano, Domenico Paparo

**Affiliations:** 1 Department of Physics, Politecnico di Milano, Piazza Leonardo da Vinci, 32, I-20133 Milano, Italy; arianna.ceraso@polimi.it; 2 INFN and Dipartimento di Fisica, Università di Roma ‘La Sapienza’, Piazzale A. Moro 2, I-00185 Roma, Italy; msengz@163.com; 3 Dipartimento di Fisica ‘E. Pancini’, Università ‘Federico II’, Monte S. Angelo, via Cintia, 80126 Napoli, Italy; rubano@fisica.unina.it; 4 ISASI, Institute of Applied Sciences and Intelligent Systems, Consiglio Nazionale delle Ricerche, via Campi Flegrei 34, 80078 Pozzuoli, Italy

**Keywords:** THz-TDS spectroscopy, nonlinear THz optics, crystalline quartz, GaSe, phonons, polaritons, THz pulse detection

## Abstract

Recently we have demonstrated a new nonlinear optical effect in the THz interval of frequencies. The latter is based on the use of femtosecond optical pulses and intense, sub-ps, broadband terahertz (THz) pulses to generate a THz-optical four- and five-wave mixing in the investigated material. The spectrum of the generated signal is resolved in time and wavelength and displays two pronounced frequency sidebands, Stokes and anti-Stokes, close to the optical second harmonic central frequency 2$\omega_{L}$, where $\omega_{L}$ is the optical central frequency of the fundamental beam, thus resembling the spectrum of standard hyper-Raman scattering, and hence we named this effect ‘THz hyper-Raman’—THYR. We applied this technique to several crystalline materials, including α-quartz and gallium selenide. In the first material, we find that the THYR technique brings spectroscopic information on a large variety of low-energy excitations that include polaritons and phonons far from the Γ-point, which are difficult to study with standard optical techniques. In the second example, we show that this new tool offers some advantages in detecting ultra-broadband THz pulses. In this paper we review these two recent results, showing the potentialities of this new THz technique.

## 1. Introduction

The Terahertz electromagnetic spectrum ranges in the frequency window from ~100 GHz to ~30 THz, i.e., in between the microwave and the far-infrared domains. The band including these frequencies was historically termed the ‘THz gap’, due to the relative difficulty of generating and detecting this elusive electromagnetic radiation. Taking advantage of the latest development of femtosecond laser technology, today available table-top THz spectrometers are becoming more and more widespread, it is now possible to generate almost single-cycle THz pulses—i.e., electromagnetic pulses with only few periods duration (~0.1 ps at 10 THz). On the other hand, a great progress for the detection of THz pulses has been achieved by the introduction of the so-called electro-optic sampling (EOS) technique, which uses part of the laser pulse generating the THz field to sample the latter. This advancement has led to the development of the so-called THz time-domain spectroscopy (THz-TDS) [1]. By means of the latter the complex optical parameters (optical conductivity, refractive index or dielectric function) of a target material are obtained by measuring the THz waveform in time-domain through EOS. Then, the recorded THz waveform is fast-Fourier-transformed in the frequency domain. Finally, the so obtained spectrum is compared with the spectrum acquired in a suitable reference sample [2,3]. By means of this procedure the real and imaginary parts of the optical parameters in the investigated spectral range are obtained without the need of Kramers–Kronig-based numerical methods [4] or in combination with the latter for improving the thickness estimation of thin films [5].

With the advent of high-power THz sources, the last decade has witnessed a rapid growth of reports on the nonlinear interaction between intense THz pulses and matter that has paved the way to the topical field of ‘THz nonlinear optics’ [6,7,8,9,10,11,12,13,14,15]. Recently we have demonstrated a new nonlinear optical effect in crystalline quartz (α-SiO_2_) that we have named THz hyper-Raman (THYR) [16]. It makes use of femtosecond infrared (IR) pulses and intense, sub-ps, broadband THz pulses to generate a four- and five-wave-mixing (FFWM) in the investigated material. The generated pulse has a central frequency at the (anti-)Stokes side of $\omega_{s;a}=2\omega_{L}\mp \omega_{T}$ for the four-wave-mixing and $\;\omega_{s;a}=2\omega_{L}\mp 2\omega_{T}$ for the five-wave-mixing, where $\omega_{L}$ and $\omega_{T}$ are the optical and THz central frequencies, respectively. The generated THYR signal is recorded as a function of both the wavelength and the temporal delay between the IR and the THz pulses.

The three-dimensional (3D) spectrum of the THYR signal may bring much information on the investigated material or on the waveform of the THz pulse. When material resonances lie in the THz band of the THYR signal these show up in the THYR 3D-spectrum as temporal oscillations. The latter may be solved in the frequency domain by applying a numerical fast Fourier transform (FFT). In the absence of resonances, the THYR 3D-spectrum may be used under some circumstances for a phase-sensitive detection of the THz optical field, as we have demonstrated in crystalline GaSe [17].

In this work we review our recent results on THYR in different crystals. In the following we will provide with a detailed description of the THYR technique, including a complete theoretical analysis, so to highlight all the potentialities of this new tool. This article is not meant to be a comprehensive review on the very recent and rapidly growing field of nonlinear optics in the THz regime. For a comprehensive review on the latter we remind to Ref. [10].

## 2. Theory

In this section we provide a detailed theory of the THYR effect. In the following derivation we will assume that the material thickness is negligible, i.e., we will not solve the Maxwell-equations for the propagating beams and we will not discuss the phase matching conditions of the process.

Let us consider two input laser pulses, each with a spectral interval of frequencies spanning frequencies $\omega_{L}$ in the visible range and $\omega_{T}$ in the THz range. Among several other mixing terms, they will induce a nonlinear polarization oscillating at the four-wave mixing frequencies $\hbar \omega_{s;a}=2\hbar \omega_{L}\mp \hbar \omega_{T}$ having an amplitude given by:

(1) $$\begin{array}{c} P_{i}^{NL}\left(\omega_{s}\right)=\;\chi_{ijhk}^{\left(3\right)}\left(2\omega_{L}-\omega_{T}\right)E_{j}\left(\omega_{L}\right)E_{h}\left(\omega_{L}\right)E_{k}^{*}\left(\omega_{T}\right)+c.c.,\\ P_{i}^{NL}\left(\omega_{a}\right)=\;\chi_{ijhk}^{\left(3\right)}\left(2\omega_{L}+\omega_{T}\right)E_{j}\left(\omega_{L}\right)E_{h}\left(\omega_{L}\right)E_{k}\left(\omega_{T}\right)+c.c., \end{array}$$

where $E^{*}$ is the complex conjugate of the field, and ${\overset{↔}{\chi }}^{\;\left(3\right)}$ is the third-order susceptibility tensor. To describe completely the THYR signal, we need to include higher order effects involving multiple THz photons. The amplitude of the optical nonlinear polarization generated in these processes (five-wave mixing) is given by:

(2) $$P_{i}^{NL}\left(\omega_{s}\right)=\;\chi_{ijhkl}^{\left(4\right)}\left(2\omega_{L}-2\omega_{T}\right)E_{j}\left(\omega_{L}\right)E_{h}\left(\omega_{L}\right)E_{k}^{*}\left(\omega_{T}\right)E_{l}^{*}\left(\omega_{T}\right)+c.c.\;P_{i}^{NL}\left(\omega_{a}\right)=\;\chi_{ijhkl}^{\left(4\right)}\left(2\omega_{L}+2\omega_{T}\right)E_{j}\left(\omega_{L}\right)E_{h}\left(\omega_{L}\right)E_{k}\left(\omega_{T}\right)E_{l}\left(\omega_{T}\right)+c.c.$$

The complex electric field of the two input laser pulses can be written:

(3) $$E_{i}\left(t\right)=\int A_{i}^{L}\left(\omega_{L}\right)e^{-i\omega_{L}t}d\omega_{L}+c.c.,\;E_{i}\left(t\right)=\int A_{i}^{T}\left(\omega_{T}\right)e^{-i\omega_{T}\left(t+\Delta t\right)}d\omega_{T}+c.c.,$$

where we have made explicit the spectral distribution for the two pulses. $A_{i}^{L,T}$ are the i-th components of the complex field amplitudes at $\omega_{L}$ and $\omega_{T}$, and Δ*t* is the temporal delay between the THz and IR pulse (for Δ*t* > 0 the THz pulse is anticipated).

### 2.1. THYR in the Presence of a Strong Second Harmonic Signal from the Fundamental Beam

According to Equations (1) and (2), the THYR optical field is proportional to the following combinations for the Stokes and anti-Stokes components, respectively:

(4) $$E^{THYR}\left(\omega_{s}\right)\propto \;\chi^{\left(3\right)}\left(2\omega_{L}-\omega_{T}\right)E_{L}^{2}\left(\omega_{L}\right)E_{T}^{*}\left(\omega_{T}\right)\;+\chi^{\left(4\right)}\left(2\omega_{L}-2\omega_{T}\right)E_{L}^{2}\left(\omega_{L}\right){\left[E_{T}^{*}\left(\omega_{T}\right)\right]}^{2}+c.c.,\;E^{THYR}\left(\omega_{a}\right)\propto \chi^{\left(3\right)}\left(2\omega_{L}+\omega_{T}\right)E_{L}^{2}\left(\omega_{L}\right)E_{T}\left(\omega_{T}\right)\;+\chi^{\left(4\right)}\left(2\omega_{L}+2\omega_{T}\right)E_{L}^{2}\left(\omega_{L}\right)E_{T}^{2}\left(\omega_{T}\right)+c.c.,$$

where, for simplicity, we have omitted any reference to the tensor and vector character of the involved quantities. In the presence of a local oscillator (LO) second harmonic signal (SHG), $E{\left(2\omega_{L}\right)}^{LO}=\chi^{\left(2\right)}E_{L}^{2}\left(\omega_{L}\right)$, generated by the fundamental beam alone and driven by the second-order constant $\chi^{\left(2\right)}$, we have interference effects that lead to the following expression for the total measured intensity.

(5) I∝ |ETHYR+ELO|2≈[ χ(2)]2IL2+[ χ(3)]2IL2IT+[ χ(4)]2IL2IT2+χ(2)χ(3) IL2Re[ET]+χ(2)χ(4) IL2Re[ET2],

where Re indicates the real part of the complex field. Moreover, for the sake of clarity, we have omitted the frequency dependence and neglected fast oscillating terms that average at zero on a THz optical cycle. $I_{L}$ and $I_{T}$ are the intensity of the fundamental and THz beam, respectively. In the latter equation we have assumed that $\;\chi^{\left(2\right)}$, $\;\chi^{\left(3\right)}$, and $\;\chi^{\left(4\right)}$ are real. This is true if we are far from material resonances, as we will show in the following for the case of GaSe, otherwise Equation (5) becomes a bit more complicated although qualitatively the same.

Given our differential detection scheme, we actually measure the following quantity

(6) ΔI≈[ χ(3)]2IL2IT+[ χ(4)]2IL2IT2+χ(2)χ(3)IL2Re[ET],

where the term ${\left[\;\chi^{\left(2\right)}\right]}^{2}I_{L}^{2}$ vanishes since it is independent on the THz field. In the latter we have also neglected the term χ(2)χ(4) IL2(ωL)Re[ET2] that is of higher order compared to χ(2)χ(3) IL2(ωL)Re[ET]. The third term of Equation (6) is interesting for the application in THz detection since it is proportional to the THz field and hence, under some circumstances, it may enable a phase-sensitive detection of the latter. When $\chi^{\left(2\right)}$ is negligibly small or the LO-SHG can be strongly reduced by using a specific geometry that spoils the optimal phase matching condition, we are left only with the terms ${\left[\;\chi^{\left(3\right)}\right]}^{2}I_{L}^{2}I_{T}$ and ${\left[\;\chi^{\left(4\right)}\right]}^{2}I_{L}^{2}I_{T}^{2}$ that allow a THYR spectroscopy of the investigated material as better explained below.

### 2.2. Semiclassical Model of THYR SPECTROSCOPY

In this section we calculated the relationship between the non-linear susceptibilities $\chi^{\;\left(3\right)}$ and $\chi^{\;\left(4\right)}$, and the macroscopic material constants that account for the induced dipole moment, the linear hyper-polarizability, and the hyper-Raman susceptibility tensor. This derivation will provide the foundation of the THYR time-domain spectroscopy (THYR-TDS).

In general, the material polarization is written:

(7) $$P_{i}\left(t\right)=\;P_{0i}+P_{Ei}=\mu_{i}+\int \alpha_{ij}A_{j}^{L}\left(\omega_{L}\right)e^{-i\omega_{L}t}d\omega_{L}+\frac{1}{2}\int \beta_{ijh}A_{j}^{L}\left(\omega_{L}\right)A_{h}^{L}\left(\omega_{L}\right)e^{-i2\omega_{L}t}d\omega_{L}+\;\int \alpha_{ij}A_{j}^{T}\left(\omega_{T}\right)e^{-i\omega_{T}\left(t+\Delta t\right)}d\omega_{T}+c.c.,$$

where the summation rule on repeated indices is assumed, and the polarization is broken in its field-dependent and non-dependent components, ${\vec{P}}_{E}$ and ${\vec{P}}_{0}$, respectively. $\mu_{i}$ is the i-th component of a possible permanent dipole of the material, $\alpha_{ij}$ is the material hyper-polarizability, and $\beta_{ijh}\;$ is the susceptibility tensor accounting for the hyper-Raman effect. In the latter equation, we have also omitted the terms of $\beta_{ijh}$ oscillating at $\omega_{L}\;\pm \;\omega_{T}$ and $2\omega_{T}$ since they eventually give rise to spectral components out of the detected range. If $\mu_{i}$, $\alpha_{ij}$, and $\beta_{ijh}$ are not constant in time, but modulated because of the variation of an oscillator material degree of freedom q(t), we can approximate the first-order variation of $P_{i}\left(t\right)$ as:

(8) $$P_{i}\left(t\right)=\;q\left(t\right)\;[\frac{\partial \mu_{i}}{\partial q}+\int \frac{\partial \alpha_{ij}}{\partial q}A_{j}^{L}e^{-i\omega_{L}t}d\omega_{L}+\int \frac{\partial \alpha_{ij}}{\partial q}A_{j}^{T}e^{-i\omega_{T}\left(t+\Delta t\right)}d\omega_{T}+\;\frac{1}{2}\int \frac{\partial \beta_{ijh}}{\partial q}A_{j}^{L}A_{h}^{L}e^{-i2\omega_{L}t}d\omega_{L}]+c.c.,$$

where we have kept only the terms proportional to q(t). We note that, even in absence of a permanent dipole moment, $\partial_{q}\mu_{i}$ may be different from zero, giving rise to a coupling with IR-active modes. The oscillator degree of freedom (i.e., the deviation from the interatomic equilibrium distance in a single ion-chain) is the solution of the classical equation for a driven damped oscillator whose solution is given by:

(9) $$q\left(t\right)=R\left(\omega_{T}\right)F\left(t\right).$$

In the latter *F*(*t*) is the driving force, while $R\left(\omega_{T}\right)$ is given by:

(10) $$R\left(\omega_{T}\right)=R_{0}+\frac{R_{\Omega }}{\Omega^{2}-\omega_{T}^{2}-2i\gamma \omega_{T}},$$

where $R_{0}$ is a possible non-resonant contribution, Ω is the proper frequency of the resonant mode, $R_{\Omega }$ is the amplitude of the resonant term, and γ is the damping constant. The driving force *F*(t) is a deterministic electromagnetic force associated with the usual dielectric energy $U=-{\vec{P}}_{0}\cdot \vec{E}-\frac{1}{2}\left({\vec{P}}_{E}\cdot \vec{E}\right)$ through the relation $F=-{\left(\partial U/\partial q\right)}_{E}$. This expression contains several terms, which are oscillating at different frequencies. Let us retain only the terms that oscillate at frequencies that are resonating with low-energy proper frequencies, i.e., terms oscillating at $\omega_{T}$ and 2$\omega_{T}$. We may then write:

(11) $$F\left(t\right)=\;\int \frac{\partial \mu_{i}}{\partial q}A_{i}^{T}e^{-i\omega_{T}\left(t+\Delta t\right)}d\omega_{T}+\frac{1}{2}\int \frac{\partial \alpha_{ij}}{\partial q}A_{i}^{T}A_{j}^{T}e^{-i2\omega_{T}\left(t+\Delta t\right)}d\omega_{T}+c.c.$$

By inserting Equation (11) in Equation (9) we obtain the modes resonant at $\omega_{T}$ and 2$\omega_{T}$:

(12) $$q\left(t\right)=\int R\left(\omega_{T}\right)\frac{\partial \mu_{i}}{\partial q}A_{i}^{T}e^{-i\omega_{T}\left(t+\Delta t\right)}d\omega_{T}+\frac{1}{2}\int R\left(2\omega_{T}\right)\frac{\partial \alpha_{ij}}{\partial q}A_{i}^{T}A_{j}^{T}e^{-i2\omega_{T}\left(t+\Delta t\right)}d\omega_{T}+c.c.$$

By replacing the expression of *q*(t) given by Equation (12) in Equation (8) we may calculate $P_{i}\left(t\right)$ and, eventually, obtain its Fourier components:

(13) $$P_{i}\left(\omega_{s},\Delta t\right)=\;\frac{R\left(\omega_{T}\right)}{4\pi }\frac{\partial \beta_{ijh}}{\partial q}\frac{\partial \mu_{k}}{\partial q}A_{j}^{L}A_{h}^{L}A_{k}^{T*}e^{-i\omega_{T}\Delta t}\delta \left(\omega_{s}-2\omega_{L}+\omega_{T}\right)+\;\frac{R\left(2\omega_{T}\right)}{4\pi }\frac{\partial \beta_{ijh}}{\partial q}\frac{\partial \alpha_{kl}}{\partial q}A_{j}^{L}A_{h}^{L}A_{k}^{T*}A_{l}^{T*}e^{-i2\omega_{T}\Delta t}\delta \left(\omega_{s}-2\omega_{L}+2\omega_{T}\right)+c.c.,$$

(14) $$P_{i}\left(\omega_{a},\Delta t\right)=\;\frac{R\left(\omega_{T}\right)}{4\pi }\frac{\partial \beta_{ijh}}{\partial q}\frac{\partial \mu_{k}}{\partial q}A_{j}^{L}A_{h}^{L}A_{k}^{T}e^{-i\omega_{T}\Delta t}\delta \left(\omega_{s}-2\omega_{L}-\omega_{T}\right)+\;\frac{R\left(2\omega_{T}\right)}{4\pi }\frac{\partial \beta_{ijh}}{\partial q}\frac{\partial \alpha_{kl}}{\partial q}A_{j}^{L}A_{h}^{L}A_{k}^{T}A_{l}^{T}e^{-i2\omega_{T}\Delta t}\delta \left(\omega_{a}-2\omega_{L}-2\omega_{T}\right)+c.c.,$$

where we have neglected terms that oscillate at frequencies around $\omega_{L}$ and not around 2$\omega_{L}.\;\delta \left(\omega \right)$ is the Dirac function that accounts for the energy conservation leading to the Stokes ($\omega_{s}$) and anti-Stokes ($\omega_{a}$) components of the spectrum. One important result of Equations (13) and (14) is the presence of the oscillatory terms $\exp\left(-i\omega_{T}\Delta t\right)$ and $\exp\left(-i2\omega_{T}\Delta t\right)$ that predict oscillations of the THYR signal as a function of the delay Δt. This prediction may be used for solving in the time-domain the material resonances, thus allowing a THYR time-domain spectroscopy (THYR-TDS) as the nonlinear analog of the THz-TDS.

Finally, by comparing Equations (1) and (2) with the latter, we obtain the following relationships for the nonlinear optical susceptibilities:

(15) $$\chi_{ijhk}^{\left(3\right)}\left(2\omega_{L}\pm \omega_{T}\right)=\;\frac{R\left(\omega_{T}\right)}{4\pi }\frac{\partial \beta_{ijh}}{\partial q}\frac{\partial \mu_{k}}{\partial q}.\;\chi_{ijhkl}^{\left(4\right)}\left(2\omega_{L}\pm 2\omega_{T}\right)=\frac{R\left(2\omega_{T}\right)}{4\pi }\frac{\partial \beta_{ijh}}{\partial q}\frac{\partial \alpha_{kl}}{\partial q}.$$

We note that $\partial_{q}\mu_{k}\neq 0$ and $\partial_{q}\alpha_{kl}\neq 0$ couple to IR- and Raman-active modes, respectively. On the other hand, $\partial_{q}\beta_{ijh}$ is a fourth-rank tensor characterized by selection rules that are different and may include those driving IR and Raman processes [18]. Therefore, the THYR technique may allow a simultaneous investigation of low-energy excitations that are complementarily present in IR and Raman spectra.

## 3. Materials and Methods 

**Figure 1:**
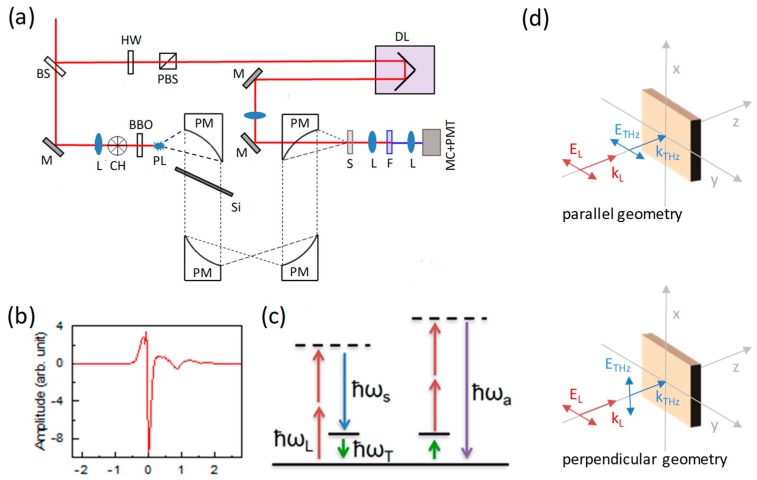
(**a**) Experimental set-up: BS—beam splitter; HW—half-waveplate; PBS—polarizing beam splitter; L—lens; M—mirror; PM—parabolic mirror; CH—triggered chopper; DL—delay line; PL—air plasma; F—colored filter; MC—monochromator; PMT—photomultiplier tube; Si—silicon plate; S—sample. (**b**) The terahertz (THz) pulse measured by means of electro-optic sampling (EOS) in LAPC. (**c**) The photon energy diagram of the THz hyper-Raman (THYR) effect. (**d**) Polarization geometry of the IR and THz beams. Upper plot for the ∥ geometry, lower plot for the ⊥ geometry.

The experimental set-up is shown in Figure 1a. A Ti: sapphire amplified laser (not shown) delivering at 1 kHz repetition rate infrared (IR) pulses of 40 fs duration, 3.5 mJ of energy, and 800 nm central wavelength, was used to generate almost-single-cycle THz pulses via the air-plasma technique [19]. The pulse time-duration was measured with standard EOS technique by means of a LAPC crystal [20] to be ~0.1 ps, as shown in Figure 1b. Although the highest frequencies of the air-plasma spectrum can be hard to detect via the EOS technique, it is commonly accepted [21] that the THz bandwidth generated with this technique is roughly ~35 THz, which is consistent with the value theoretically expected for a 40 fs laser bandwidth. In addition, the presence of a thick silicon filter in the THz path ensures that frequencies higher than ~35 THz would be strongly dumped by Si-O-Si vibrational modes. The IR and THz beams were linearly polarized. The polarization of the THz beam could be adjusted by means of a pair of wire-grid polarizer.

The THz beam was collimated and focused into the crystal under investigation by 90° off-axis parabolic mirrors with a 4 inch focal length. The output THYR signal was filtered to remove the strong residual of the fundamental laser beam by means of a 3-mm-thick Schott-BG39 filter. The filtered THYR signal was then dispersed by a blazed angle grating to analyze its spectral components separately. The signal was thus measured as a function of the THz-IR time-delay Δ*t* with a time step equal to 0.02 ps and as a function of the wavelength with a wavelength step equal to 0.5 nm. We implemented a differential detection scheme, thus the THYR signal was measured as the difference between a THz-ON and a THz-OFF subsequent pulses by means of a mechanical chopper, which cut every second pulse on the THz line. The laser repetition rate was 1 kHz, so that the THz pulse had a repetition rate of 500 Hz. When the LO-SHG was not zero, this detection scheme allowed us to remove the signal coming from the first term of Equation (5) so that we were left only with the signal depending on the THz field and its intensity, i.e., ΔI of Equation (6).

Several commercial α-SiO2 samples of different axes orientation (X-, Y- and Z-cut) and thicknesses (50, 500 and 1000 µm) were measured. The signal was found to be vanishing in all Z-cut samples. All the samples show qualitatively comparable results, although with some quantitative differences probably due to the phase matching conditions that strongly depends on sample thickness. In all the data presented here we focused our attention on the intermediate sample of 500 µm, while a detailed comparison among all the samples would be the subject of a forthcoming publication. By rotating the THz and IR polarizations with respect to the α-SiO_2_ optical axis Z, all possible combinations were investigated. We found a non-vanishing signal for only two cases: Z || IR || THz and Z || IR ⊥ THz, where the Z optic axis was set parallel to the y-axis of the laboratory frame. For simplicity we will refer to these two cases with the labels ‘parallel’ || ($\Delta I_{\left|\right|}$) and ‘perpendicular’ ⊥ ($\Delta I_{⊥}$), respectively. The polarization geometries are reported in detail in Figure 1d. For the experiments on GaSe, we used a commercial 30 µm-thick Z-cut crystal. In this case the polarization geometry was kept fixed to the parallel one. Both crystalline quartz and GaSe were non-centrosymmetric crystals and hence they displayed a significant SHG contribution to the signal. The latter could be made negligible or could be enhanced by varying the phase-matching conditions. This depends on the use of THYR for a spectroscopic investigation or for the detection of THz pulse, respectively. In the case of quartz, we set the optical axis Z always parallel to the IR polarization. According to the selection rules in quartz, SHG should vanish in this geometry. We were able to make SHG negligibly small, although a small residual was already present probably due to quadrupolar contributions. On the contrary, in GaSe the LO-SHG was made significantly different from zero.

## 4. Results

**Figure 2:**
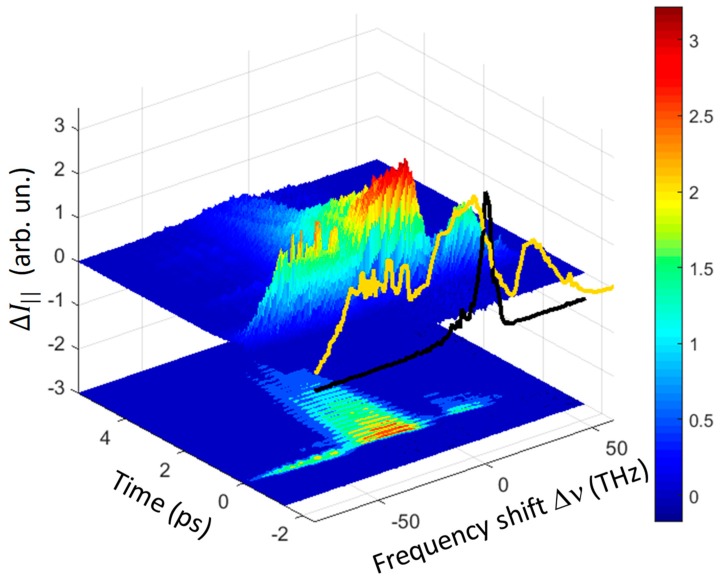
3D diagram of the THYR signal for the || polarization combination in a quartz crystal as a function of time and frequency shift Δν. A cut of the signal at *t* = 0 is reported in front of the diagram (yellow curve) together with the LO-SHG spectrum (black curve). Below the 3D diagram a contour plot of it is reported, highlighting the signal oscillations lasting for about 4 ps.

**Figure 3:**
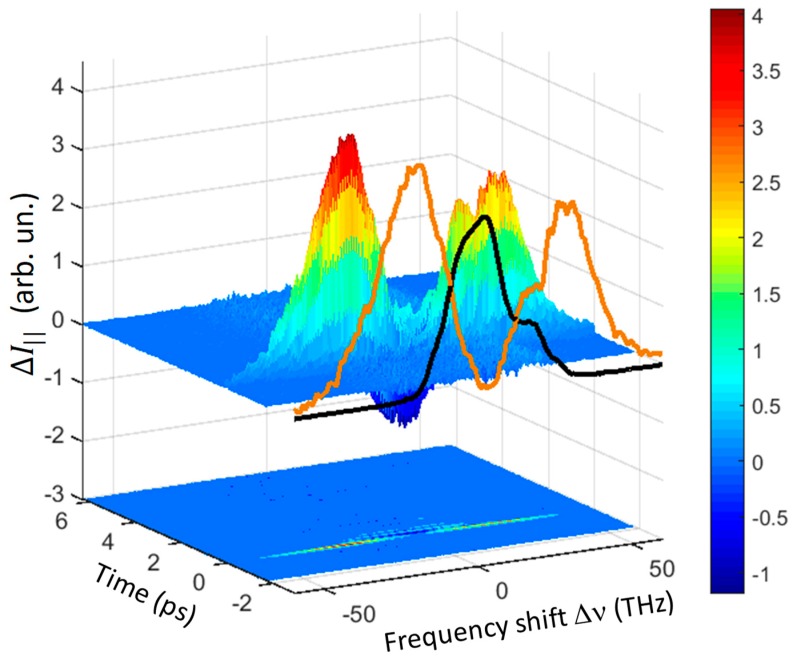
3D diagram of the THYR signal for the || polarization combination in a GaSe crystal as a function of time and frequency shift Δν. A cut of the signal around *t* = 0 is reported in front of the diagram (orange curve) together with the LO-SHG spectrum (black curve). Below the 3D diagram a contour plot of it is reported. Note the absence of long lasting oscillations, as opposed to quartz.

The main results of our experiments are well summarized in Figure 2 and Figure 3, where the three-dimensional (3D) spectra measured for the || geometry in quartz and GaSe are shown. The THYR signal was reported as a function of the time delay Δt and the frequency shift Δν from the second-harmonic central frequency that corresponds to a wavelength of about 400 nm. A negative frequency shift corresponds to a Stokes component of the spectrum, while a positive shift indicates an anti-Stokes component. In each figure a projection of the signal around time zero is reported in front of the 3D diagrams (yellow curve in Figure 2 and orange curve in Figure 3). In these 2D projections, the spectrum of the LO-SHG was included (black curve). A contour plot of the 3D graphs is reported at the bottom of each figure.

In both materials it was evident that the observed signal spanned a wide spectral range going from about −80 to +50 THz. It is worth noting that the Stokes wing extended up to about 80 THz, which was almost twice the band estimated for the THz pulse. A so large bandwidth might be explained only by considering four-wave-mixing and even five-wave-mixing processes, involving one or two THz photons, as explained in Section 2. These spectra were composed by three distinct bands (going from right to left): (i) ‘first-order anti-Stokes band’ (ASB1, 0–40 THz), (ii) ‘first-order Stokes band’ (SB1, 0–−40 THz) and (iii) ‘second-order Stokes band’ (SB2, −40–−80 THz). One additional band, the ‘second-order anti-Stokes band’ (ASB2, ~40–80 THz) might be seen (data not shown) by replacing the filters in order to allow the shortest wavelength to be detected.

A second fundamental feature of these spectra was the oscillatory behavior of the signal in time as highlighted by the contour plot of Figure 2. A significant amount of signal was still present for Δt > 1 ps, i.e., an order of magnitude larger than the THz pulse duration. This delayed signal appeared however mainly in the SB1 band, while the ASB1 band had a much shorter decay and the SB2 and ASB2 bands present an almost instantaneous response. It is also worth noting that in the THYR spectrum of quartz the term χ(2)χ(3) IL2Re[ET] was negligibly small since the phase matching condition was set so to minimize the LO-SHG. Therefore the observed oscillations might be ascribed solely to material resonances as discussed in Section 2.2.

Interestingly, these strong oscillations were not observed in the case of GaSe, as shown in the contour plot of Figure 3. In the latter case the THYR signal decayed almost instantaneously. At zero time, $\Delta I_{∥}$ displayed two positive peaks in the Stokes and anti-Stokes regions of the spectrum. At the central frequency a negative dip appeared at the latest time (see the blue dip in the 3D plot of Figure 3). The latter was more evident in the time-cut shown in Figure 7a that would be discussed in detail below. In the absence of strong material oscillations, the negative dip was due to the interference between the LO-SHG and the THYR signal. These interference effects were almost absent in the Stokes and anti-Stokes regions of the spectrum. This was a consequence of the reduced overlap of the Stokes and anti-Stokes spectra with the LO-SHG spectrum. It is also worth noting that the anti-Stokes peak was lower than the Stokes one. This is not surprising in general since the $\;\chi^{\left(3\right)}$ and $\;\chi^{\left(4\right)}$ material constants strongly depend on the wavelength [22]. However, in our case, this difference found a simple explanation in the reduced transmittivity of the BG39 filter in the anti-Stokes range.

**Figure 4:**
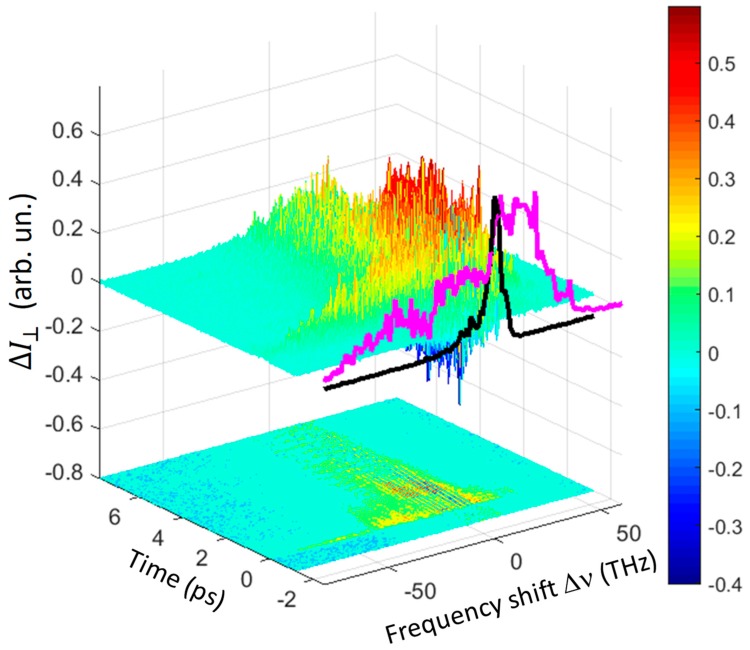
3D diagram of the THYR signal for the ⊥ polarization combination in a quartz crystal as a function of time and frequency shift Δν. A cut of the signal around *t* = 0 is reported in front of the diagram (purple curve) together with the LO-SHG spectrum (black curve). Below the 3D diagram a contour plot of it is reported, highlighting the oscillations that last for more than five ps. Note the larger signal in the anti-Stokes interval as compared to the || geometry (Figure 2).

Going back to quartz, it is worth noting that the ΔI  signal was strongly dependent on the polarization geometry. This is exemplarily shown in Figure 4, where the 3D spectrum for the ⊥ polarization combination is reported. As highlighted in the projection of the spectrum at time zero, the THYR signal was more enhanced on the anti-Stokes side of the spectrum as compared to the || geometry. This is not surprising since the phase-matching conditions for the FFWM processes strongly depend on the polarizations of the mixed beams.

**Figure 5:**
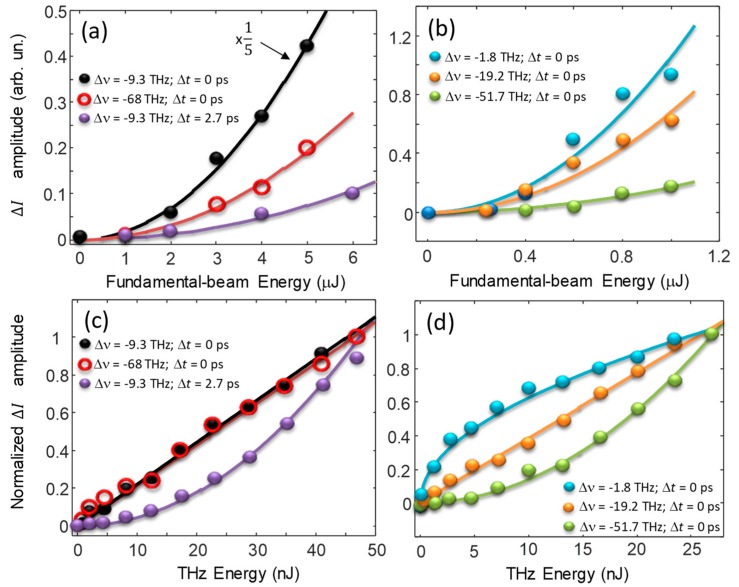
(**a**,**b**) THYR peak signal for different frequency shifts as a function of the fundamental-beam energy for quartz and GaSe, respectively. The geometry is the parallel one and the THz pulse energy is set at 20 nJ. Panel (**a**): energy dependence for Δν = −9.3 THz at zero (black solid circles) and 2.7 ps (red open circles) time delay, and for Δν = −68 THz at zero delay (purple solid circles). Note the factor of five for the signal represented by black solid circles. Panel (**b**): energy dependence at zero delay for Δν = −1.8 THz (cyan solid circles), for Δν = −19.2 THz (orange solid circles), and for Δν= −51.7 THz (green solid circles). (**c**,**d**) Normalized THYR peak for different frequency shifts as a function of the THz energy for quartz and GaSe, respectively. The IR energy is set at 2 μJ. The meaning of the symbols is the same as above.

To further confirm the model described by Equation (6), the ΔI signal at t = 0 was measured as a function of both the THz and IR pulse energy. In the case of quartz, where the signal displays a longer decay time, these measurements were repeated at a different delay time too. The results are shown in Figure 5. As a first observation, we note that the ΔI intensity peak scales with the square of the fundamental beam intensity for all the Δν considered here (panel (a) and (b) of Figure 5). This result confirms the prediction of Equation (6) where each term depends on $I_{L}^{2}$, i.e., the square of the fundamental beam intensity or equivalently the square of pulse energy. In quartz the same behavior might be observed also at latest times, as shown in Figure 5a for a time delay of 2.7 ps (red solid line). It is worth noting that the measurement at latest times could be performed only at −9.3 THz since at −68 THz the signal decay was quite instantaneous.

The behavior of ΔI as a function of the THz pulse-energy was different between quartz and GaSe. This is because, in the case of GaSe, all the three terms of Equation (6) were different from zero. Let us first focus on quartz, whose results are displayed in panel (c) of Figure 5.

The THYR signal scaled linearly with the THz pulse energy at −9.3 THz, i.e., within the band SB1, while it scaled quadratically at −68 THz, which was at the extreme of the SB2 band. These results confirm that the SB1 band results from FFWM processes involving a single THz photon as described by the term ${\left[\;\chi^{\left(3\right)}\right]}^{2}I_{L}^{2}I_{T}$ of Equation (6), while the SB2 band originates from two-THz-photon processes described by the term ${\left[\;\chi^{\left(4\right)}\right]}^{2}I_{L}^{2}I_{T}^{2}$. At the frequency shift of −9.3 THz the linear dependence on $I_{T}$ might be observed at latest times too (red solid line in Figure 5c).

Analogous considerations might be drawn in the case of GaSe. At the frequency shift of −19.2 THz a linear dependence on $I_{T}$ was observed (single-THz-photon process), while at −51.7 THz the dependence on $I_{T}$ was quadratic (two-THz-photon process). As a difference with the case of quartz, around the central frequency, the third term of Equation (6) was present and dominated in this spectral region. The latter was proportional to the amplitude of the THz optical field, which in turn was proportional to the square-root of the THz pulse intensity or energy. This behavior as a function of the THz pulse energy was nicely confirmed by the orange solid line of Figure 5d, which was a fit obtained by using a squared-root function.

## 5. Discussion

### 5.1. THYR as a Spectroscopic Tool

**Figure 6:**
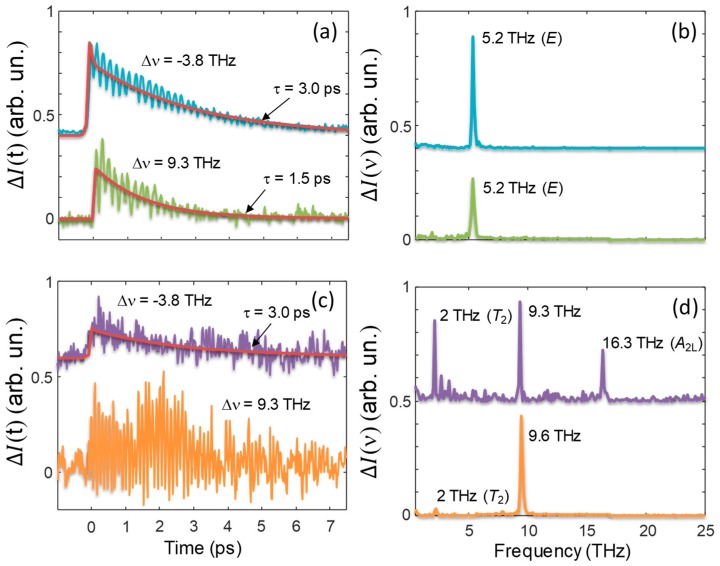
(**a**,**c**) Behavior of the THYR signal from quartz as a function of the delay Δt for different frequency shift, and for the || and ⊥ geometry, respectively. Red curves are best-fits of the non-resonant contribution. The corresponding decay times are reported by each curve. (**b**,**d**) Corresponding spectra obtained by means of FFT after subtraction of the non-resonant background. The labels close to each peak indicate the value of the resonance frequency and its symmetry when available.

In this section we will show how the diagrams of Figure 2 and Figure 4 may be used for obtaining spectroscopic information on the material under investigation. We will limit here only to a few examples for illustrating how the technique works while we refer the reader to reference [16] for a more detailed analysis. Due to the large laser and THz bandwidths, it is not possible to resolve the THYR signal in the frequency domain. Nonetheless, by taking advantage of the same principle at work in THz time-domain spectroscopy, it is possible to follow the evolution of the THYR signal in time and access the frequency domain again by FFT. To do this we need to perform time-cuts of the 3D graph at different frequency shifts. Two of these time-cuts are shown exemplarily in Figure 6a,c for the || and ⊥ geometry, respectively.

In Figure 6a we always observed a pronounced peak at Δt = 0 ps followed by an exponential decay. For the ⊥ geometry, the non-resonant contribution was only distinguishable for Δυ= −3.8  THz. When the non-resonant contribution was significant, we fit the data with a function that was the sum of two contributions: a Gaussian, which accounts for the instantaneous processes at Δt = 0 ps, corresponding to the signal commonly considered in THz field-induced SHG (TFISH) [23,24,25,26,27], and a single-exponential decay, whose decay time τ represents the relaxation time of the non-resonant term. The resulting fit functions of this ‘non-resonant’ part of the THYR signal are reported as red solid curve in Figure 6a,c. The obtained values of τ are reported in Figure 6 by each curve. It is interesting to note that at Δυ=−3.8  THz we had the same value of τ independently from the polarization geometry. The presence of this non-resonant decay could be likely ascribed to a big number of acoustic phonons created by the strong electric field of the THz pulse via the piezoelectric effect. We did not discuss this specific aspect in further detail here, as it would be addressed elsewhere.

More interesting than the non-resonant part of the THYR signal is, of course, the ‘resonant part’, which appears as distinguished oscillations superimposed to the decay. The latter are dominant in Figure 6c where a non-resonant contribution could be observed only at Δυ=−3.8  THz. To study the frequency spectrum of these oscillations, when meaningful, we subtracted the fit-function discussed above from the data and finally applied a FFT. The results are displayed in panel (b) and (d) of Figure 6 for the || and ⊥ geometry, respectively. In order to interpret the measured spectra, let us now compare our results with the literature, and in particular Table I of reference [28], where they report the frequency and the symmetry of the phonon modes observed in quartz. In Figure 6 we have reported the symmetry of these modes near the corresponding resonance, when this assignment is possible. A closer inspection of Figure 6 allowed us to highlight the following features of the THYR spectroscopy:

1. According to reference [28] the ***A*_2*L*_** mode at 16.3 THz is IR-only active and hence cannot be observed with standard Raman spectroscopy. In our case the selection rules followed those of the hyper-Raman susceptibility ***β_ijh_*** (see Equation (15)) that were different from those driving IR and Raman processes. Therefore IR-active modes were visible in the THYR spectrum.
2. The mode ***T*_2_** at 2 THz and the mode *E* at 5.2 THz belong to the M and A points of the Brillouin Zone, respectively. Usually, optical spectroscopies cannot detect modes far from the Γ point, because of the very small momentum of optical photons, which can induce only vertical transitions, and therefore affects mainly the states close to the central Γ point. However, if two phonons are simultaneously created with opposite momenta, the process is allowed. Therefore, in our THYR spectra there were evidences of these two-phonon excitations. It is worth noting that two-phonon processes are usually accompanied by resonances that are less sharp than the ones shown in Figure 6, since several phonons from different branches may combine at once. Indeed, in the 1-mm-thick sample and at different frequency shifts (data not shown), we observed that the resonance at 2 THz displayed a larger width. This is probably the result of a complex dependence on the phase matching conditions that might suppress or enhance some modes. This issue deserves to be investigated more in detail in the future.
3. The mode around 9.5 THz cannot be assigned directly to one of the listed phonon modes [28]. The closest one is a phonon at 10.7 THz with an ***A*_2_** symmetry. In our previous work we have demonstrated that the resonance at 9.5 THz may be assigned to a polaritonic mode generated by the coupling of light with the ***A*_2_** phonon [16].

The previous list shows the richness of information carried by the THYR spectra.

### 5.2. Application of THYR for the Detection of THz Pulses

In the presence of a strong LO signal the term $\chi^{\left(2\right)}\chi^{\left(3\right)}\;I_{L}^{2}R\left[E_{T}\right]$ of Equation (6) dominates in the central part of the spectrum, where the overlap between $\chi^{\left(2\right)}$ and $\chi^{\left(3\right)}$ is maximum. In this case, by performing time-cut around the central frequency, the THYR signal may be used for a phase-sensitive detection of the THz optical field. At this aim it is important to investigate if the waveform of this term might be distorted by the presence of strong oscillations due to the material lattice resonances, as those observed in the crystalline quartz. This information can be obtained by analyzing the full spectrum of the THYR signal as shown in Figure 3. As already discussed, it was evident from the latter figure that in the investigated THz range there were no strong oscillations lasting for times longer than the pulse duration. Therefore we expected that the waveform extracted by means of a time-cut around the central frequency carried mostly information on the THz optical field. We note that the waveform obtained in this way was analogous to that measured by means of TFISH. However, as already remarked, the THYR technique was more informative and included TFISH since it provides with a full characterization of the material spectral properties. This is the main strength of the THYR technique as compared to the TFISH technique.

**Figure 7:**
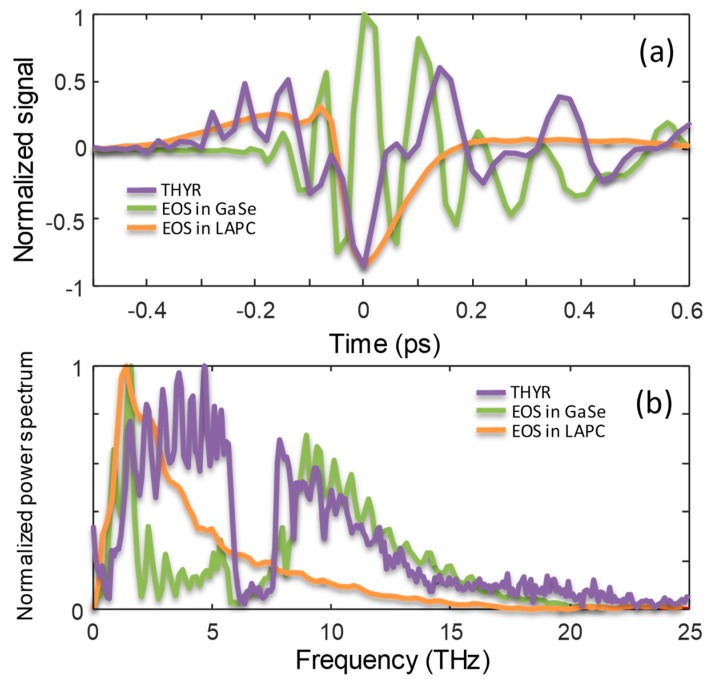
(**a**) The THz pulse waveform as measured by means of THYR (purple solid line), and EOS in GaSe (green solid line) and LAPC (orange solid line). The waveforms have been normalized to their amplitude. (**b**) Corresponding normalized power spectra.

The THYR time-cut is reported in Figure 7a together with two waveforms obtained by means of EOS in the GaSe crystal itself and a LAPC crystal [20], for comparison. In Figure 7b the corresponding power spectra are shown. The latter demonstrates that THYR in GaSe allowed us to detect pulses with a bandwidth up to 25 THz. We observed that our technique was more efficient than the standard EOS in GaSe for detecting the signal components in the frequency interval from about 2.5 to 7.5 THz, and a bit more also above 20 THz. This shows that the THYR signal was less affected by the material resonances at the lowest frequencies, as anticipated by the previous analysis of the THYR 3D spectrum. This is a consequence of the different phase-matching rules driving the two optical processes in GaSe, i.e., EOS and THYR.

The LAPC detection was smoother over all the range and more efficient in the range 6–8 THz. However, LAPC had a reduced bandwidth that did not allow an efficient detection of frequencies above 17 THz. The higher efficiency of the THYR technique in these two spectral intervals was reflected also in the temporal waveforms. Given the large suppression of the lowest frequencies, the EOS signal in GaSe shows multiple oscillations during the duration of the single cycle of the THYR signal and the EOS signal in LAPC. On the other hand, the single cycle of the latter had a larger duration than the THYR pulse since the EOS in LAPC cut off the highest frequencies.

## 6. Conclusions

In this paper we reviewed our recent results on a new nonlinear optical effect in the THz interval of frequencies, which we named THYR. With two examples we showed how this effect might be used for providing spectroscopic information on the material under investigation or for a phase sensitive detection of a THz pulse.

In the first example, where we investigated single crystals of quartz, our THYR spectra bring information on a large variety of low-energy excitations including polaritons and phonons far from the Γ-point. The latter were generally difficult to study with standard optical techniques, including Raman and THz-TDS spectroscopy. Therefore, our new THYR spectroscopy demonstrated to be highly complementary to other standard spectroscopic techniques.

In the second example, where we observed strong interference effects of the THYR signal with a second-harmonic local oscillator, we found that the THYR technique allowed a more efficient detection of the lowest spectral components and of the frequencies above 20 THz of a THz pulse as compared to EOS measurements in two popular crystals. Thus, the new technique shows some advantages for the detection of broadband THz pulses as compared to other detection schemes.

In conclusion, we believe that our THYR technique significantly expands the potential of THz nonlinear optics and spectroscopy that in the last two decades has already become an invaluable tool in pulse detection, sensing, spectroscopy and many fields of material science and testing.
